# Squamoid eccrine ductal carcinoma of the left thumb: A case report and review of treatment strategies

**DOI:** 10.1016/j.jdcr.2025.08.024

**Published:** 2025-09-10

**Authors:** Alyssa M. Iurillo, Victoria Hoffman, Neha Kinariwalla, Jaclyn B. Anderson, Karen Nevarez, Leslie Robinson-Bostom, Tiffany J. Libby, Thomas J. Miner, Mohamedyak A. Puthawala, Oliver Wisco

**Affiliations:** a Department of Dermatology, Indiana University School of Medicine, Indianapolis, Indiana; b Department of Dermatology, Jacobs School of Medicine and Biomedical Sciences at the University of Buffalo, Buffalo, New York; c Department of Dermatology, The Warren Alpert Medical School of Brown University, Providence, Rhode Island; d Department of Pathology, The Warren Alpert Medical School of Brown University, Providence, Rhode Island; e Department of Surgical Oncology, The Warren Alpert Medical School of Brown University, Providence, Rhode Island; f Department of Radiation Oncology, The Warren Alpert Medical School of Brown University, Providence, Rhode Island

**Keywords:** metastasis, perineural invasion, radiation, SEDC, SLNB, squamoid eccrine ductal carcinoma, surgery, XRT

## Introduction

Squamoid eccrine ductal carcinoma (SEDC) is a rare malignant adnexal tumor of sweat gland origin, characterized by aggressive local invasion and a significant risk of recurrence.^1^ Since 1991, when it was first described,^2^ a total of 68 cases have been reported in the literature. We report a case of SEDC and review the literature to provide guidance on diagnosis, prognosis, and management. Due to its rarity, there is sparse literature and agreement on the standard of care. As such, treatment approaches are often adapted from experience with similar cutaneous adnexal neoplasms. Early detection and a multidisciplinary approach are crucial to optimizing patient outcomes, as delayed diagnosis and incomplete excision increase the likelihood of recurrence and metastasis.^3^

Histopathologically, SEDC exhibits squamoid features with perineural invasion (PNI) and may mimic squamous cell carcinoma (SCC), necessitating immunohistochemical analysis for accurate diagnosis.^4^ Treatment options vary from wide local excision and Mohs micrographic surgery (MMS), to amputation in cases with high-risk features. Given the risk of recurrence and PNI, adjuvant radiation therapy (XRT) is often considered to enhance local control.^5^

This report presents a case of SEDC of the left thumb in a 78-year-old female patient, detailing the diagnostic challenges, multidisciplinary treatment strategy, and outcomes, as well as a review of current treatment modalities.

## Case report

**Fig 1:**
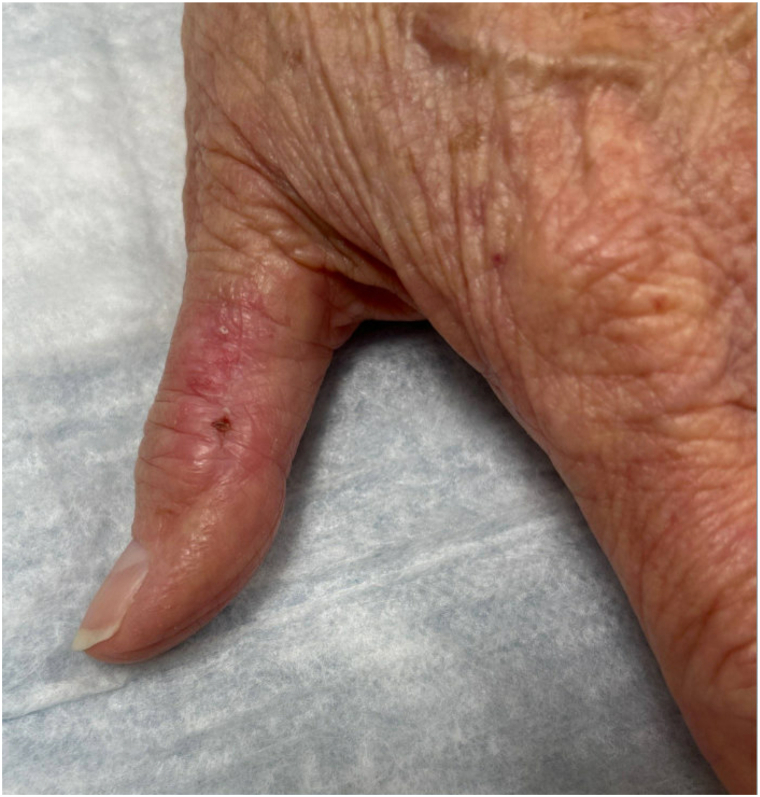
A 5-mm macule on left thumb. No treatment done.

**Fig 2:**
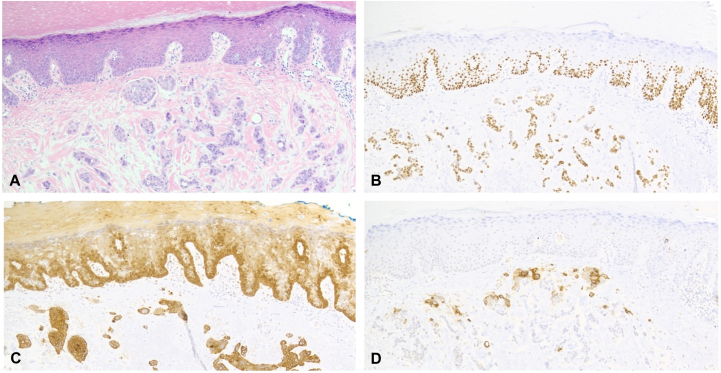
Pathology images.

A 78-year-old right-handed female patient with a history of stage 3B chronic kidney disease and hypertension initially presented to orthopedics with a 3-year history of left thumb pain. On examination, she had a 5-mm nonblanching purpuric macule with surrounding blotchy erythema on her left thumb (Fig 1). The patient underwent an excisional biopsy, and histology showed squamoid nests and cords infiltrating through a highly hyalinized dermis. Occasional foci of nests contained vague duct and lumen formation. PNI (nerve diameter of 0.05 mm) was present. The overlying epidermis showed scattered keratinocyte atypia throughout, suggestive of an overlying carcinoma in situ. Immunohistochemistry demonstrated that the lesional cells were diffusely positive for CK5/6 and p63, whereas carcinoembryonic antigen highlighted the scattered ductal differentiation. The lesional cells were negative for CK7, S100, and ER. D2-40 immunostain failed to highlight foci of lymphovascular invasion (Fig 2).

The case was sent to the multidisciplinary tumor board for review for discussion of MMS surgery versus amputation and sentinel lymph node biopsy (SLNB) for staging. The board recommended SLNB, MMS surgery, and hand reconstruction, with adjuvant XRT planned postoperatively. Given the risk of metastasis, lymph node evaluation was prioritized. Medical oncology deferred recommendations until lymph node results were available. Radiation oncology suggested adjuvant therapy for high-risk features such as PNI.

**Fig 3:**
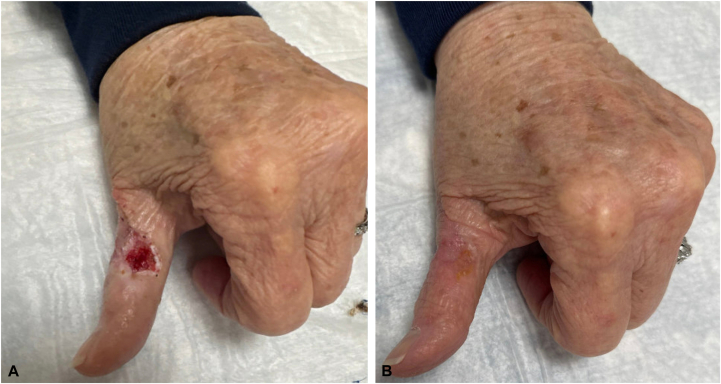
Left thumb after biodegradable temporizing matrix removal at follow-up plastic surgery appointment **(A)**. One month later at a follow-up appointment **(B)**.

Surgical oncology performed an SLNB of 2 left axillary lymph nodes, both of which are negative for metastasis. The patient underwent MMS with clear margins in a single stage. Plastic surgery then reconstructed the defect through an adjacent tissue transfer with a tissue matrix, which was later removed at follow-up (Fig 3). The patient's postoperative course was uncomplicated, and she consistently used a splint. Pathology confirmed clear MMS margins, with no residual tumor at the deep or lateral edges.

This case highlights the rarity and complexity of SEDC management, requiring a multidisciplinary approach. The patient successfully underwent SLNB, MMS, and hand reconstruction, with clear margins and no lymph node involvement. Adjuvant XRT is currently planned twice a week for 5 weeks using brachytherapy, totaling 10 fractions.

## Discussion

SEDC presents significant challenges due to its propensity for local recurrence and regional spread. Current literature suggests that wide local excision is the most commonly used treatment (86% of cases); however, it carries a recurrence rate of up to 26%.^6^ MMS has demonstrated improved margin control in cutaneous malignancies and, in this case, was the preferred approach to optimize physical function while ensuring oncologic safety.^7^

A key consideration was the role of SLNB given the estimated 13% risk of metastasis in SEDC.^8^ SLNB was performed in this patient, with negative results, guiding the decision to proceed with MMS rather than amputation. Studies suggest regional lymph node evaluation may be warranted for high-risk features, particularly PNI or large tumor size.^9^

XRT treatment planning was critical, and with clear final margins achieved via MMS, the immediate need for radiation was negated. Future adjuvant XRT is planned due to PNI and literature supporting its use in SEDC with neurovascular invasion, multiple failed excisions, or recurrence. However, limited data prevent a full assessment of benefits, as recurrence and disease progression-related mortality have been reported.^10^^,^^11^ Long-term surveillance is crucial due to the risk of delayed recurrence in adnexal malignancies.^12^

Distinguishing between SEDC and SCC poses diagnostic challenges due to their overlapping histologic features, including squamoid morphology, infiltrative growth, and PNI. Immunohistochemical staining is critical for diagnosis, as SEDC typically expresses epithelial membrane antigen, carcinoembryonic antigen, and cytokeratins CK5/6 and p63.^4^^,^^13^ Negative expression of these markers (CK5/6 and p63) has not been reported in SEDC.^13^ Most cases of SEDC have an epidermal connection, with no in situ component or one that appears distinct from SCC in situ.^14^ Although sampling tissue, if a superficial shave or punch biopsy is done, that only captures the surface squamoid appearance, the tissue could be indistinguishable from a well or moderately differentiated SCC, which is reported in previously published cases.^14^^,^^15^ Overall, immunohistochemical staining clarifies the diagnosis; carcinoembryonic antigen and epithelial membrane antigen highlight ductal differentiation in SEDC, whereas a similar staining pattern is not expected in SCC.^14^

**Fig 4:**
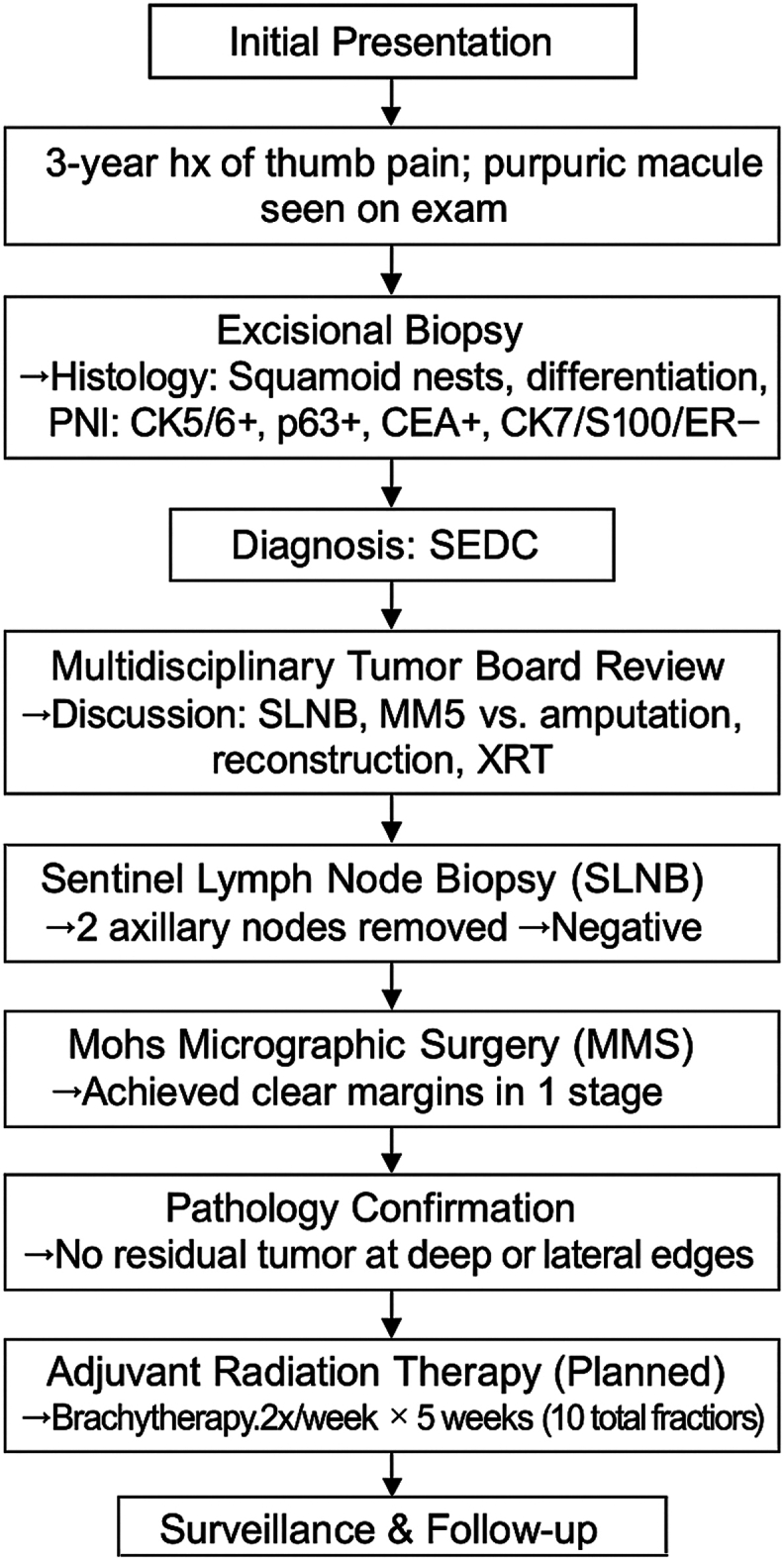
Management flow diagram for a thumb squamoid eccrine ductal carcinoma. *CEA*, Carcinoembryonic antigen; *PNI,* perineural invasion; *SEDC*, squamoid eccrine ductal carcinoma; *XRT,* adjuvant radiation therapy.

This case underscores the importance of a multidisciplinary approach, balancing oncologic control with functional preservation in digital tumors. Collaboration among dermatology, dermatopathology, surgical oncology, plastic surgery, and radiation oncology was essential in determining the most appropriate treatment plan. The management timeline is summarized in a flow diagram for clarity (Fig 4). The use of MMS over amputation highlights the evolving approach to rare adnexal malignancies, where maximizing oncologic outcomes must be carefully weighed against maintaining quality of life.

## Conflict of interest

None disclosed.
